# New application of intelligent agents in sporadic amyotrophic lateral sclerosis identifies unexpected specific genetic background

**DOI:** 10.1186/1471-2105-9-254

**Published:** 2008-05-30

**Authors:** Silvana Penco, Massimo Buscema, Maria Cristina Patrosso, Alessandro Marocchi, Enzo Grossi

**Affiliations:** 1 Medical Genetics, Clinical Chemistry and Clinical Pathology Laboratory, Niguarda Ca' Granda Hospital P.za Ospedale Maggiore 3, 20100 Milan, Italy; 2 Semeion Research Center Via Sersale 117, 00128 Rome, Italy; 3 Bracco SpA Medical Department Via E. Folli 50, 20134 Milan, Italy

## Abstract

**Background:**

Few genetic factors predisposing to the sporadic form of amyotrophic lateral sclerosis (ALS) have been identified, but the pathology itself seems to be a true multifactorial disease in which complex interactions between environmental and genetic susceptibility factors take place. The purpose of this study was to approach genetic data with an innovative statistical method such as artificial neural networks to identify a possible genetic background predisposing to the disease. A DNA multiarray panel was applied to genotype more than 60 polymorphisms within 35 genes selected from pathways of lipid and homocysteine metabolism, regulation of blood pressure, coagulation, inflammation, cellular adhesion and matrix integrity, in 54 sporadic ALS patients and 208 controls. Advanced intelligent systems based on novel coupling of artificial neural networks and evolutionary algorithms have been applied. The results obtained have been compared with those derived from the use of standard neural networks and classical statistical analysis

**Results:**

Advanced intelligent systems based on novel coupling of artificial neural networks and evolutionary algorithms have been applied. The results obtained have been compared with those derived from the use of standard neural networks and classical statistical analysis. An unexpected discovery of a strong genetic background in sporadic ALS using a DNA multiarray panel and analytical processing of the data with advanced artificial neural networks was found. The predictive accuracy obtained with Linear Discriminant Analysis and Standard Artificial Neural Networks ranged from 70% to 79% (average 75.31%) and from 69.1 to 86.2% (average 76.6%) respectively. The corresponding value obtained with Advanced Intelligent Systems reached an average of 96.0% (range 94.4 to 97.6%). This latter approach allowed the identification of seven genetic variants essential to differentiate cases from controls: apolipoprotein E arg158cys; hepatic lipase -480 C/T; endothelial nitric oxide synthase 690 C/T and glu298asp; vitamin K-dependent coagulation factor seven arg353glu, glycoprotein Ia/IIa 873 G/A and E-selectin ser128arg.

**Conclusion:**

This study provides an alternative and reliable method to approach complex diseases. Indeed, the application of a novel artificial intelligence-based method offers a new insight into genetic markers of sporadic ALS pointing out the existence of a strong genetic background.

## Background

Amyotrophic lateral sclerosis (ALS), the most common form of motoneuron disease, is a relatively rare (incidence: 1–3/100.000 per year), progressive and fatal disease characterised by neurodegeneration involving primarily motor neurons of the cerebral cortex, brain stem and spinal cord. To date, most studies have focused upon the familial form of the disease, which accounts for less then 10% of cases, and which is usually inherited as autosomal dominant inheritance. The gene coding for copper/zinc superoxide dismutase 1 (SOD1) appears to be mutated in 10–20% in the familial form [1].

Genetic risk factors for ALS have been extensively studied and some "major genes", in addition to SOD1, have been recognised as being responsible for the monogenic inheritance pattern. There are now at least six dominant inherited adult onset ALS genes of which only three have been identified so far [2]. However, most ALS cases seem to be a typical multifactorial disease deriving from the interaction between a number of genes and environmental factors, some of which are still not established as causing of the disease, including brain and spinal cord trauma, strenuous physical activity, exposure to radiation [3].

Current hypotheses suggest a complex interplay between multiple mechanisms including genetic risk factors, oxidative stress, neuroexcitatory toxicity, mitochondrial dysfunction, intermediate neurofilament disorganization, failure of intracellular mineral homeostasis involving zinc, copper, or calcium, disrupted axonal transport, abnormal protein aggregation or folding, and neuroinflammation [3,4]. Recently there has been growing interest in the role played by non-neuronal neighbourhood cells in the pathogenesis of motor neuron injury and in the dysfunction of specific molecular signal pathways [5,6].

Among the genetic factors that may predispose to sporadic ALS, neurofilaments, apolipoprotein epsilon 4 genotype, excitotoxicity genes, ciliary neurotrophic factor (CTNF), cytochrome P450 debrisoquine hydroxylase CYP2D6, apurinic apyrimidinic endonuclease (APEX), mitochondrial manganese superoxide dismutase SOD2, monoamine oxidase allele B and paraoxonases, have been reported in different studies, partly with contradictory results [2,4,7-9]. Not all the published studies have been replicated, probably because of the different populations analysed as well as insufficient sample size. On the other hand, different studies have employed either tissue microdissection or microarray technologies to search for other "low penetrant" or "susceptibility" genes that are more common in the population and often polymorphic and the combination and interaction of these with environmental factors may contribute to modulate individual risk [10-12]. Recently, several genome-wide association studies have been performed with innovative approaches, i.e. the Illumina platform, and the authors have identified SNPs (single nucleotide polymorphisms) potentially associated with ALS [13-16]. However most genome-wide association studies have not confidently identified risk genes that are replicated in every study. The most likely causes are disease heterogeneity, allelic heterogeneity, small effect sizes and probably, insufficient sample size. However, so far no microarrays panel has been specifically developed for ALS and the aetiology of the disease still remains to be defined.

Some years ago our group had the opportunity of working on another multifactorial complex disease such as venous thrombosis and analysing the results by an innovative statistical approach, Artificial Neural Networks (ANNs) [17]. Indeed, ANNs promises to improve the predictive value of traditional statistical data analysis. Initially, a known set of data, from a given problem with a known solution, is learned by the ANNs and subsequently the networks can reconstruct the fuzzy rules which may be underlying a complex set of data. ANNs have been successfully used in many areas of medicine as recently illustrated in an extensive review by Lisboa [18], as well as by Ritchie et al [19] where neural networks were used for supervised pattern recognition in genetic epidemiology, and also in SNPs association studies [20-22]. Much effort has been spent to adapt ANNs architectures and the ensembles to specific problems to be solved. More specifically many novel computational approaches have been developed and applied with special attention to complex gene-gene, gene-environment interactions and ANNs [19-23].

The literature data together with the impressive results we obtained with ANNs, by which we were able to identify a subset of polymorphisms related to the disease, prompted us to employ the same approach also in ALS hoping to discover specific genetic patterns underlying the sporadic form of this disease. We applied a multiarray approach including allelic variations in genes that could be involved in the pathogenesis of ALS disease since it has been demonstrated that inflammation, cellular adhesion, and lipid pathways are linked to such a disease [10,11]. On the contrary, it has never been demonstrated that regulation of blood pressure, coagulation, homocysteine metabolism and matrix integrity pathways are directly linked to ALS even though they could be indirectly.

Genotyping of ALS cases and controls was performed. We applied advanced intelligent systems based on novel coupling of artificial neural networks and evolutionary algorithms and compared the results with those obtained by linear discriminant analysis and a simple back propagation approach.

Surprisingly, we discovered a novel strong genetic background allowing a correct classification of cases and controls with a higher than 90% accuracy.

## Methods

### Subjects

The study population included subjects of Caucasian origin belonging to Italian ancestry and consisted of 54 sporadic ALS (SALS) patients and 208 control subjects.

Diagnostic Criteria for ALS disease were based on the World Federation of Neurology El Escorial Revisited document [24]. All patients diagnosed to have Definite, Probable or Probably laboratory-supported ALS, who gave their informed consent, were included in the study. The diagnosis of Possible ALS was also accepted. According to common clinical practice, our cases were subdivided into bulbar and spinal onset on the basis of the first symptoms reported by each patient. All patients, referred to the Department of Neurology of Niguarda Hospital, Milan from 2001 to 2005, were defined sporadic when the disease was present in a single member of the family and when no mutations were present in SOD1 gene.

Control subjects were selected from a healthy control population, randomly collected from healthy blood donors admitted to the "Healthy Blood Donor Service" of Niguarda Ca' Granda Hospital. We checked the absence of personal and familial history of ALS in this group through direct interview.

This study was approved by the local ethics committee.

### Genotyping

DNA was extracted using a salting out procedures [25]. We applied a multilocus assay, as previously described [17,26], to genotype 60 biallelic polymorphisms within 35 genes that were selected from pathways of lipid and homocysteine metabolism, regulation of blood pressure, coagulation, inflammation, cellular adhesion and matrix integrity. The following polymorphisms (SNPs) were genotyped: LPA 93C/T, 121 G/A, APOA4 thr347ser, glu360his, APOBthr71ile, APOC3 641C/A, 482C/T, 455 T/C, 1100 C/T, 3175 C/G, 3206 T/G, APOE cys112arg, arg158cys, ADRB3 trp64arg, PPARγ pro12ala, LIPC 480C/T, LPL 93 T/G, asp9asn, asn291ser, ser447term, PON1 met55leu, gln192arg, PON2 ser311cys, LDLR NcoI+/-, CETP-631C/A, -629 C/A, ile405val, TNF beta thr26asn, MTHFR 677 C/T, NOS3 -922 A/G, -690 C/T, glu298asp, DCP1 IVS16 ins/del, AGTR1 1166A/C, AGT met235thr, NPPA 664 G/A, NPPA 2238 T/C, ADD1 gly460trp, SCNN1A trp493arg, ala663thr, GNB3 825 C/T, ADRB2 arg16gly, ADRB2 gln27glu, MMP3 (-1171) 5A/6A, FII 20210 G/A, FV arg506gln, FVII -230 10 bp del/ins, arg353glu, PAI -675 G5/G4, 11053 G/T, FGB -455 G/A, ITGA2 873 G/A, ITGB3 leu33pro, SELE ser128arg, leu554phe, ICAM gly214arg, TNF alpha -376 G/A, -308G/A, -244 G/A, -238 G/A.

The marker TNF beta thr26asn is twice present in the arrays as a control for the multiplex PCR and the hybridization procedures.

All ALS subjects were screened for SOD1 mutation through PCR amplification and direct sequencing according to standard procedures [27].

### Database

Each record related to a known clinical condition or to a sample population, and comprised 62 variables corresponding to the 60 SNPs plus case and control. We eliminated from the database those markers for which only one genotype was present (APOB Arg3500Gln, CBS Ile278Thr, CETP Asp442Gly, 14G(+1) A and 14(+3) T ins) both in cases and controls. All the analysed polymorphisms may have three genotype classes: wild type, heterozygous and homozygous status. The association of these variables with ALS status was tested by ANNs and the results were compared with those obtained by a linear discriminant analysis. The models we used aimed at correct classification of the subjects in two classes:

1) SALS patients (cases),

2) healthy subjects (controls).

No other specific genetic model potentially linked to the analysed SNP was evaluated; ANNs are able to build a model with a strong genetic basis just collecting all the information included within the SNP without any *a priori *definition. The mathematical approach of ANNs consists in measuring the general dependence of random variables related to a group of subject without making any assumption about the nature of their underlying relationships.

### Artificial neural networks analysis

In this study we applied supervised ANNs, in order to develop a model able to predict with high degree of accuracy the diagnostic class starting from genotype data alone.

Supervised ANNs are networks which learn by examples, calculating an error function during the training phase and adjusting the connection strengths in order to minimize the error function. The learning constraint of the supervised ANNs make their own output coincide with the predefined target. The general form of these ANNs is: y = f(x,w*), where w* constitutes the set of parameters which best approximate the function.

We employed the **Back Propagation (BP) **ANNs [28]. This type of ANN belongs to a very large family of ANNs, that normally uses a specific kind of law of learning named Feed Forward (FF). In the FF ANNs the signal proceeds from the input to the output of the ANN, crossing all of the nodes once only. The architecture of these networks is characterized by different layers of interconnected nodes (input, hidden and output nodes), which processes the input signal according to a non-linear function (generally, of sigmoid type). The fundamental equation that characterizes the activation of a single node and, therefore, the signal transfer from one layer to another is:

$$x_{j}^{\left[s\right]}=f\left(\sum_{i=0}^{n}w_{ji}^{\left[s\right]}\cdot x_{i}^{\left[s-1\right]}\right)$$

Learning, i.e. the modelling of the input-output relation represented by data, occurs through minimization of the error in output and retropropagation of this to the internal nodes, one hidden units, using the algorithm of the descending gradient in the majority of cases. In particular each weight is corrected by the formula:

$$\Delta w_{ji}^{\left[s\right]}=-lcoef\cdot \frac{\partial \;E}{\partial \;w_{ji}^{\left[s\right]}}=lcoef\cdot e_{j}^{\left[s\right]}\cdot x_{i}^{\left[s-1\right]}$$

where for the retropropagated error $e_{j}^{\left[s\right]}$ we have:

$$e_{j}^{\left[out\right]}=f^{'}\left(I_{j}^{\left[out\right]}\right)\cdot \left(t_{j}-x_{j}^{\left[out\right]}\right)$$

for the last layer and:

$$e_{j}^{\left[s\right]}=f^{'}\left(I_{j}^{\left[s\right]}\right)\cdot \sum_{k}\left(e_{k}^{\left[s+1\right]}\cdot w_{kj}^{\left[s+1\right]}\right)$$

for all the other layers.

In theory, a Back Propagation having a sufficient number of hidden units is able to reconstruct any **y = f(x) **function.

The BP used in this work was intentionally improved through the use of the SoftMax equation, specific for classification problems [29]:

$$y_{k}=\frac{e^{I_{k}}}{\sum_{l=1}^{k}e^{I_{l}}}$$

and through the use of the Selfmomentum equation [30] which appears as follows:

$$\Delta w_{ji}^{\left[s\right]}\left(n\right)=lcoef\cdot e_{j}^{\left[s\right]}\left(n\right)\cdot x_{i}^{\left[s-1\right]}\left(n\right)+Selfmomentum_{ji}\left(n\right)$$

$$Selfmomentum_{ji}\left(n\right)=\left|e_{j}^{\left[s\right]}\left(n-1\right)\right|\cdot \frac{1}{0.5+\left|w_{ji}^{\left[s\right]}\left(n-1\right)\right|}\Delta w_{ji}^{\left[s\right]}\left(n-1\right)$$

where the learning cycle is indicated by *n*.

From a practical point of view, the Selfmomentum equation allows the solution of all of the problems solved by the Momentum, in a much faster way, maintaining the unitary learning coefficient (Rate = 1).

The architecture of ANN BP-FF is an input layer according to the number of selected variables, one hidden layer according to the different input layer (min 2 nodes, max 12 nodes). The output layer consisting in one of two prediction targets (SALS cases; control).

We employed as benchmark linear discriminant analysis (LDA) applied on the same training and testing data sets used for ANNs. For the analysis of LDA, the SAS version 6.04 (SAS Institute, Cary, NC, USA) using forward stepwise procedure was employed.

### Preprocessing methods and experimental protocols

Data preprocessing was performed using two different re-sampling criteria of the global dataset.

#### - Random criterion

We employed the so-called 5 × 2 cross-validation protocol [31]. In this procedure the study sample is five-times randomly divided into two sub-samples, always different but containing similar distribution of cases and controls: the training one (containing the dependent variable) and the testing one. During the training phase the ANNs learn a model of data distribution and then, on the basis of such a model, classify subjects in the testing set in a blind way. Training and testing sets are then reversed and consequently 10 analyses for every model employed are conducted.

#### -Optimized criterion: TWIST system

The TWIST system is an ensemble of two algorithms: "Training and Testing" (T&T) and "Imput Selection" (I.S.) algorithm [32].

The T&T system is a robust data resampling technique that is able to arrange the source sample into sub-samples that all possess a similar probability density function. In this way, the data is split into two or more sub-samples in order to train, test and validate the ANN models more effectively. The T&T is based on a population of n ANNs managed by an evolutionary system. In its simplest form, this algorithm reproduces several distribution models of the complete dataset DΓ (one for every ANN of the population) in two subsets ($d_{\Gamma }^{\left[tr\right]}$, the Training Set and $d_{\Gamma }^{\left[ts\right]}$, the Testing Set). During the learning process each ANN, according to its own data distribution model, is trained on the subsample $d_{\Gamma }^{\left[tr\right]}$ and blind-validated on the subsample $d_{\Gamma }^{\left[ts\right]}$.

The performance score reached by each ANN in the testing phase represents its "fitness" value (i.e., the individual probability of evolution). The genome of each "network-individual" thus codifies a data distribution model with an associated validation strategy. The n data distribution models are combined according to their fitness criteria using an evolutionary algorithm. The selection of "network-individuals" based on fitness determines the evolution of the population; that is, the progressive improvement of performance of each network until the optimal performance is reached, which is equivalent to the better division of the global dataset into subsets. The evolutionary algorithm mastering this process, named "Genetic Doping Algorithm" (GenD) (33) has similar characteristics to a genetic algorithm but it's able to maintain an inner instability during the evolution, carrying out a natural increase of biodiversity and a continuous "evolution of the evolution" in the population. The elaboration of T&T is articulated in two phases:

- preliminary phase: in this phase an evaluation of the parameters of the fitness function that will be used on the global dataset is performed. During this phase an inductor $\Omega_{D_{\Gamma }^{\left[tr\right]},A,F,Z}\left(\cdot \right)$ is configured, which consists of an ANN with an algorithm (A) Back Propagation standard. For this inductor the optimal configuration to reach the convergence is stabilized at the end of different training trials on the global dataset *D*_Γ_; in this way the configuration that most "suits" the available dataset is determined: the number of layers and hidden units and some possible generalizations of the standard learning law. The parameters thus determined define the configuration and the initialization of all the individual-networks of the population and will then stay fixed in the following computational phase. Basically, during this preliminary phase there is a fine-tuning of the inductor that defines the fitness values of the population's individuals during evolution.

The accuracy of the ANN performance with the testing set will be the fitness of that individual (that is, of that hypothesis of distribution into two halves of the whole dataset).

- Computational phase: the system extracts from the global dataset the best training and testing sets. During this phase the individual-network of the population is running, according to the established configuration and the initialization parameters. From the evolution of the population, managed by the GenD algorithm, the best distribution of the global dataset D Γ into two subsets is generated, starting from the initial population of possible solutions $x=\left(D_{\Gamma }^{\left[tr\right]},D_{\Gamma }^{\left[ts\right]}\right)$. Preliminary experimental sessions are performed using several different initialization and configuration of the network in order to achieve the best partition of the global dataset.

Parallel to T&T runs I.S. The IS system is an adaptive system, which is also based on the evolutionary algorithm GenD, and which is able to evaluate the relevance of the different variables of the dataset in an intelligent way. Therefore it can be considered on the same level as a feature selection technique. From a formal point of view, I.S. is an artificial organism based on the GenD algorithm and consists of a population of ANN, in which each one carries out a selection of the independent variables on the available database. The elaboration of I.S., as for T&T, is developed in two phases:

- Preliminary phase: during this phase an inductor $\Omega_{D_{\Gamma }^{\left[tr\right]},A,F,Z}\left(\cdot \right)$ is configured to evaluate the parameters of the fitness function. This inductor is a standard Back-Propagation ANN. The parameters configuration and the initialization of the ANNs are carried out with particular care to avoid possible over-fitting problems that can be present when the database is characterized by a high number of variables that describe a low quantity of data. The number of epochs *E*_0 _necessary to train the inductor is determined through preliminary experimental tests.

- Computational phase: the inductor is active, according to the stabilized configuration and the fixed initialization parameters, to extract the most relevant variables of the training and testing subsets. Each individual-network of the population is trained on the training set $D{'}_{\Gamma }^{\left[tr\right]}$ and tested on the testing set $D{'}_{\Gamma }^{\left[ts\right]}$.

The evolution of the individual-network of the population is based on the algorithm GenD. In the I.S. approach the GenD genome is built by n binary values, where n is the cardinality of the original input space. Every gene indicates if an input variable is to be used or not during the evaluation of the population fitness. Through the evolutionary algorithm, the different "hypotheses" of variable selection, generated by each ANNs of the population, change over time, at each generation: this leads to the selection of the best combination of input variables. As in the T&T systems the genetic operators crossover and mutation are applied on the ANNs population; the rates of occurrence for both operators are self-determinated by the system in adaptive way at each generation.

When the evolutionary algorithm no longer improves its performance, the process stops, and the best selection of the input variables is employed on the testing subset.

In order to improve the speed and the quality of the solutions that have to be optimized, the GenD algorithm makes the evolutionary process of the artificial populations more natural and less centered on the individual liberalism culture.

The combined action of T&T and I.S. systems allow us to solve two frequent problems in managing ANNs. Both systems are based on a Genetic Algorithm, the Genetic Doping Algorithm (GenD) developed at Semeion Research Centre [33].

GenD was provided with 100 individuals, generated randomly. Each individual represents a possible distribution of the whole dataset into two subsets. Two independent Multilayers Perceptrons (MLPs) with 4 hidden units, are trained for 100 epochs and tested in blinded manner on the two subsets. A function of the testing results of the two independent MLPs defines the fitness of each individual.

A crossover function is applied on the populations of 100 individuals and new individuals are generated. A mutation operator is applied to the new individuals and to the individuals whose fitness is weakest. In the GenD algorithm the rate of crossover and the rate of mutation are self-determined by the system in adaptive way at each generation. This loop is applied for at least 300 generations, or stopped when the system does not show any significant improvement at least from 50 generations. The individual whose distribution of the whole dataset into two subsets is the best one from the blind testing results is saved and then used as optimal distribution to train and test more sophisticated ANNs.

We implemented both algorithms in C language and we used a Pentium III CPU to run the system on real data. Around 48 hours were spent for each run. We remind that T&T and I.S. algorithms have to be used only once to train the system. Once trained, the system can answer on line to any new pattern.

After this processing, the features that were most significant for the classification were selected and at the same time the training set and the testing set were created with a function of probability distribution similar to the one that provided the best results in the classification.

A supervised Multi Layer Perceptron, with four hidden units, was then used for the classification task.

## Results

### Study populations

We collected 54 patients (mean age at onset of disease 59.62 years; range 53.7 – 65.5 years): 28 males (56.4 years; 46.9 – 65.8) and 26 females (62.9 years; 57.8 – 67.9) with a male/female ratio 1.1:1. The site of clinical onset was spinal in 61.1% (33/54) and bulbar in 38.9% (21/54) of cases. The mean disease duration at the time of observation was 3.2 years (range 1–10 years). The frequency of bulbar onset in females (16/29) resulted greater than in males (5/28).

All patients were previously screened for SOD1 gene mutation by sequence analysis and no genetic variations were found.

Control subjects were 144 males and 67 females; age range 21 to 75 years, (average 38.94).

### Genotyping analysis

Table 1 summarizes the distribution of the SNPs in the two groups of patients and controls. The reliability of the whole molecular procedure (multiplex and hybridization steps) was checked by the TNF beta thr26asn polymorphism that gave the same results in both strips A and B for the same subject analyzed (see 17 and 26 for details).

**Table 1:** Genotype distribution at each marker locus: wild type (WT); heterozygous type (Hetero); homozygous type (Homo).

		**Cases: 54**			**Controls: 208**				
					
		**Frequency**							
					
	**rs number**	**WT**	**Homo**	**Hetero**	**WT**	**Homo**	**Hetero**	**f (A)**	**f (a)**
LPA 93 C/T	rs1853021	45	0	9	161	6	41	0,872596	0,127404
LPA 121 G/A	rs1800769	33	1	20	166	3	39	0,891827	0,108173
APOA4 thr347ser	rs675	32	1	21	135	8	65	0,805288	0,194712
APOA4 gln360his	rs5110	44	0	10	183	1	24	0,9375	0,0625
APOB thr71ile	rs1367117	35	5	14	100	20	88	0,692308	0,307692
APOC3 -641C/A	rs2542052	17	6	31	74	21	113	0,627404	0,372596
APOC3 -482 C/T	rs2854117	23	2	29	104	13	91	0,71875	0,28125
APOC3 -455 T/C	rs2854116	16	7	31	74	24	110	0,620192	0,379808
APOC3 1100 C/T	rs4520	30	3	21	104	24	80	0,692308	0,307692
APOC3 3175 C/G	rs5128	47	1	6	170	2	36	0,903846	0,096154
APOC3 3206 T/G	rs4225	21	13	20	72	37	99	0,584135	0,415865
APOE cys112arg	rs429358	49	0	5	175	1	32	0,918269	0,081731
APOE arg158cys	rs7412	45	1	8	186	1	21	0,944712	0,055288
ADRB3 trp64arg	rs4994	45	0	9	185	0	23	0,944712	0,055288
PPAR pro12ala	rs1801282	48	0	6	170	1	37	0,90625	0,09375
LIPC -480 C/T	rs1800588	29	9	16	146	11	51	0,824519	0,175481
LPL -93 T/G	rs1800590	54	0	0	201	0	7	0,983173	0,016827
LPL asp9asn	rs1801177	54	0	0	201	0	7	0,983173	0,016827
LPL asn291ser	rs268	53	0	1	202	0	6	0,985577	0,014423
LPL ser447term	rs328	47	0	7	164	3	41	0,887019	0,112981
PON1 met55leu	rs854560	2	19	33	31	84	93	0,372596	0,627404
PON1 gln192arg	rs662	26	10	18	102	26	80	0,682692	0,317308
PON2 ser311cys	rs6954345	33	2	19	131	6	71	0,800481	0,199519
LDLR NcoI +/-	rs5742911	28	5	21	98	19	91	0,689904	0,310096
CETP -631 C/A	rs1800776	45	0	9	168	2	38	0,899038	0,100962
CETP -629 C/A	rs1800775	6	16	32	49	60	99	0,473558	0,526442
CETP ile405val	rs5882	20	9	25	86	30	92	0,634615	0,365385
TNFbeta thr26asn	rs1041981	31	7	16	113	12	83	0,742788	0,257212
MTHFR 677 C/T	rs1801133	19	12	23	60	35	113	0,560096	0,439904
NOS3 -922 A/G	rs1800779	9	11	34	57	52	99	0,512019	0,487981
NOS3 -690 C/T	rs3918226	40	2	12	164	3	41	0,887019	0,112981
NOS3 glu298asp	rs1799983	18	11	25	75	36	97	0,59375	0,40625
DCP1 IVS16 ins/del	rs1799752	24	8	22	38	70	100	0,423077	0,576923
AGTR1 1166 A/C	rs5186	31	5	18	99	10	99	0,713942	0,286058
AGT met235thr	rs699	14	4	36	67	46	95	0,550481	0,449519
NPPA 664 G/A	rs5063	52	0	2	194	0	14	0,966346	0,033654
NPPA 2238 T/C	rs5065	31	3	20	149	3	56	0,850962	0,149038
ADD1 gly460trp	rs4961	36	3	15	145	4	59	0,838942	0,161058
SCNN1A trp493arg	rs5742912	54	0	0	186	0	22	0,947115	0,052885
SCNN1A ala663thr	rs2228576	27	3	24	94	18	96	0,682692	0,317308
GNB3 825 C/T	rs5443	30	5	19	103	19	86	0,701923	0,298077
ADRB2 arg16gly	rs1042713	11	21	22	31	84	93	0,372596	0,627404
ADRB2 gln27glu	rs1042714	24	7	23	89	22	97	0,661058	0,338942
MMP3(-1171) 5A/6A	rs3025058	9	14	31	34	59	115	0,439904	0,560096
FII 20210 G/A	rs1799963	53	0	1	200	0	8	0,980769	0,019231
FV arg506gln	rs6025	53	0	1	204	0	4	0,990385	0,009615
FVII del/ins	rs5742910	41	1	12	144	4	60	0,836538	0,163462
FVII arg353gln	rs6046	40	1	13	152	3	53	0,858173	0,141827
PAI (-675) 5G/4G	rs1799768	8	21	25	40	52	116	0,471154	0,528846
PAI 11053 G/T	rs7242	13	12	29	39	66	103	0,435096	0,564904
FGB -455 G/A	rs1800790	34	2	18	121	8	79	0,771635	0,228365
ITGA2 873 G/A	rs1062535	22	7	25	72	27	109	0,608173	0,391827
ITGB3 leu33pro	rs5918	37	1	16	159	6	43	0,867788	0,132212
SELE ser128arg	rs5361	45	0	9	168	2	38	0,899038	0,100962
SELE leu554phe	rs5355	47	0	7	180	1	27	0,930288	0,069712
ICAM gly214arg	rs1799969	54	0	0	171	0	37	0,911058	0,088942
TNF alpha-376 G/A	rs1800750	51	0	3	204	0	4	0,990385	0,009615
TNF alpha-308 G/A	rs1800629	44	0	10	158	1	49	0,877404	0,122596
TNF alpha-244 G/A	rs673	54	0	0	207	0	1	0,997596	0,002404
TNF alpha-238 G/A	rs361525	52	0	2	192	0	16	0,961538	0,038462

Deviations of the genotype frequencies from the Hardy-Weinberg equilibrium were tested in the control group with chi-squared statistics (*p *values ranging from 0.2 to 1.0). Allele frequencies at each marker locus were calculated from the genotype frequencies of the control group under the null hypothesis of Hardy-Weinberg equilibrium. Allele frequencies at each marker locus are reported.

### Classification performances with ANNs

Results obtained with Linear Discriminant Analysis were compared with those obtained with a simple Back Propagation approach (Table 2 and 3).

**Table 2:** Results obtained applying the random criterion and classifying with the linear discriminant analysis.

**LDA**	**ALS**	**Controls**	**A. Mean Acc**	**W. Mean Acc**	**Errors**
LDA(1ab)	78%	78%	78%	78%	29
LDA(1ba)	93%	70%	81%	74%	33
LDA(2ab)	82%	78%	80%	79%	28
LDA(2ba)	82%	79%	80%	79%	27
LDA(3ab)	63%	81%	72%	77%	30
LDA(3ba)	74%	74%	74%	74%	34
LDA(4ab)	78%	71%	75%	73%	36
LDA(4ba)	67%	81%	74%	78%	29
LDA(5ab)	82%	67%	75%	70%	39
LDA(5ba)	59%	73%	66%	70%	39

**Mean**	**76%**	**75%**	**75%**	**75%**	**32**

The second and third columns report the percentage of patients correctly classified as belonging to cases or controls. The fourth and fifth columns report the accuracy obtained by the model as arithmetic mean and weighted mean. The number of errors is reported in the last column. The last row reports the mean values of all the columns.

**Table 3:** Results obtained applying the random criterion and classifying with a simple back propagation.

**ANN**	**ALS**	**Controls**	**A. Mean Acc**	**W. Mean Acc**	**Errors**
FF_Bp(1ab)	74%	85%	79%	82%	23
FF_Bp(1ba)	89%	84%	86%	85%	20
FF_Bp(2ab)	82%	83%	82%	82%	23
FF_Bp(2ba)	78%	83%	80%	82%	24
FF_Bp(3ab)	56%	94%	75%	86%	18
FF_Bp(3ba)	63%	88%	76%	83%	22
FF_Bp(4ab)	67%	84%	75%	80%	26
FF_Bp(4ba)	59%	85%	72%	80%	26
FF_Bp(5ab)	63%	80%	71%	76%	31
FF_Bp(5ba)	52%	86%	69%	79%	27

**Mean**	**68%**	**85%**	**77%**	**82%**	**24**

The second and third columns report the percentage of patients correctly classified as belonging to cases or controls. The fourth and fifth columns report the accuracy obtained by the model as arithmetic mean and weighted mean. The number of errors is reported in the last column. The last row reports the mean values of all the columns.

In these experiments we applied the random criterion to divide the dataset five times in training and testing sub-sets applying the 5 × 2 Cross Validation protocol.

The predictive accuracy obtained with Linear Discriminant Analysis and standard artificial neural networks ranged from 70% to 79% (average 75.31%) and from 69.1 to 86.2% (average 76.6%) respectively.

With the TWIST approach, every experiment was conducted in a blind and independent manner in two directions: training with sub-sample A and blind testing with sub-sample B vs training with sub-sample B and blind testing with sub-sample A. The results from the best five applications of TWIST procedures are reported in Table 4. This advanced intelligent system, through the final selection of a subgroup of 25–27 variables along ten independent applications, provided the highest predictive performance with a sensitivity ranging from 92.0% to 100% (average 96.75%), and a specificity ranging from 91.67% to 98.81% (average 95.78%) and with an overall accuracy ranging from 94.4 to 97.6% (average 96.0%). In all the TWIST system experiments the 90% overall accuracy threshold was exceeded whereas Back Propagation and Linear Discriminant Analysis never exceeded the 80% threshold.

**Table 4:** Results of ten experiment obtained applying TWIST procedure in an independent manner to the whole dataset.

**Experiment**	**ALS**	**Controls**	**A. Mean Acc**	**W. Mean Acc**	**Errors**
TWIST 1ab	96%	97%	96.5%	96%	6
TWIST 1ba	98%	95%	97.5%	96%	4
TWIST 2 ab	100%	94%	97%	96%	5
TWIST 2 ba	100%	96%	98%	96%	5
TWIST 3 ab	97%	99%	98%	97%	4
TWIST 3 ba	95%	99%	97%	96%	3
TWIST 4 ab	98%	90%	95%	93%	9
TWIST 4 ba	100%	94%	97%	94%	7
TWIST 5 ab	92%	96%	94%	96%	5
TWIST 5 ab	92%	98%	96%	96%	5

**Mean**	**97%**	**96%**	**96%**	**96%**	**5**

In the first column ab means training on subset a and testing on subset b; ba means the opposite. The second and third columns report the percentage of patients correctly classified as belonging to cases or controls. The fourth and fifth columns report the accuracy obtained by the model as arithmetic mean and weighted mean. The number of errors is reported in the last column. The last row reports the mean values of all the columns.

#### Genetic variants selected by the five TWIST procedures

Seven genetic variants were always independently selected by the five TWIST procedures: apolipoprotein E (APOE) (chromosome 19q13.2) arg158cys; hepatic lipase (LIPC) (chromosome 15q21-23) -480 C/T; endothelial nitric oxide synthase (NOS3) (chromosome 7q36) 690 C/T and glu298asp; vitamin K-dependent coagulation factor seven (F7) (chromosome 13q34) arg353glu, glycoprotein Ia/IIa (ITGA2) (chromosome 5q23-q31) 873 G/A; E-selectin (SELE) (chromosome 1q22-q25) ser128arg.

Table 5 gives the results obtained with ANNs using only the input data derived from these variants.

**Table 5:** Results obtained with ANNs using only the seven genetic variants selected by TWIST procedure.

**ANN**	**ALS**	**Control**	**A. Mean Acc**	**W. Mean Acc**	**Errors**
FF_Bp(ab)	93%	87%	90%	88%	16
FF_Bp(ba)	89%	74%	82%	78%	28

**Mean**	91%	81%	86%	83%	22

The second and third columns report the percentage of patients correctly classified as belonging to cases or controls. The fourth and fifth columns report the accuracy obtained by the model as arithmetic mean and weighted mean. The number of errors is reported in the last column. The last row reports the mean values of all the columns.

#### Genetic variants independently selected by four TWIST procedures

The number of genetic variants selected four times over five experiments consisted of: peroxisome proliferator activated receptor gamma (PPARG) pro12ala (chromosome 3p25), lipoprotein lipase (LPL) asp9asn (chromosome 8p22), paraoxonase 1 (PON1) met55leu and paraoxonase 2 (PON2) ser311cys (chromosome 7q21.3), tumor necrosis factor beta (TNF beta) thr26 asn (chrom 6p21.3), methylenetetrahydrofolate reductase (MTHFR) 677 C/T (chrom 1p36.3), angiotensin II receptor type1 (AGTR1) 1166 A/C (chromosome 3q21-25), atrial natriuretic peptide (NPPA) 664 G/A (chrom 1p36-21), epithelial Na channel subunit (SCNN1A) trp493arg, (chromosome 12p13), FVII -232 ins/del, SELE leu554phe, Tumor Necrosis Factor alpha (TNFalpha) -376 G/A and -308 G/A (chromosome 6p21.3).

The TNF beta thr26asn was used as further control. First it was selected by four TWIST systems and later, since the information linked to such a variation was already recruited, none of the TWIST systems selected this SNP.

#### Genetic variants never selected by any TWIST procedures

The following gene/genetic variants were never selected by the five TWIST procedures: apolipoprotein A4 (APOA4) (chromosome 11q23) thr347ser; apolipoprotein C3 (APOC3) (chromosome 11q23.1-q23.2) -641 C/A and 482 C/T; beta 3 adrenergic receptor (ADRB3) trp64arg (8p12-p11.2); LPL ser447term; PON1 gln192arg; low density lipoprotein receptor (LDLR) (chromosome 19p13.3) exon 18 NcoI +/-; cholesteryl ester transfer protein (CETP) -631 C/A and -629 C/A (chromosome 16q21); NOS3 922 A/G; G-protein beta 3 subunit (GNB3) 825 C/T (chromosome 12p13); beta 2 adrenergic receptor (ADBR2) arg16gly (chromosome 5q31-q32); beta fibrinogen (FGB) -455 G/A (chromosome 4q28); TNF alfa -238 G/A and TNF beta thr26asn.

## Discussion

The mechanism of neurodegeneration in ALS remains an enigma. The major problem is that little is known about the disease mechanism, making candidate gene selection difficult and haphazard. It follows that an unconventional approach of making no a priori assumptions about the location of the variants of interest might be appropriate, provided that a similarly unconventional statistical approach is available to manage the data complexity.

Comparison of results obtained using three different analytical approaches (classical statistics, standard neural networks and advanced artificial neural networks), points out the need to employ systems that are really able of handling the disease complexity instead of treating the data with reductionist approaches unable to detect multiple genes of smaller effect in predisposing to the disease. The possibility of obtaining high diagnostic accuracy from limited and selected genetic information using these new analytical tools, shows that even in so-called sporadic ALS the genetic background plays a fundamental role.

Another important obstacle in approaching the molecular basis of a rare disease like ALS in a conventional manner, is related to the difficulty of finding a homogeneous sample population large enough to be analysed for a wide number of genetic variants. Artificial neural networks, at variance with the classical statistical tests, can manage complexity even with relatively small samples and the subsequent unbalanced ratio between variables and records. In this connection, it is important to note that adaptive learning algorithms of inference, based on the principle of a functional estimation like artificial neural networks, overcome the problem of dimensionality.

Internal validation of the prediction accuracy is one of the most important problems in neural networks analysis. In fact, the restriction of training procedures to only a part of the dataset, generally half of it, causes a potential loss of power to recognize hidden patterns. In this study optimization of the training and testing procedures were addressed using the evolutionary training and testing algorithm, which ensured that the two halves of the dataset contained the same amount of relevant information. Thus, the best division of the whole dataset into a training and a testing set was reached after a finite number of generations. Finally ANNs were able to identify gene combinations (allelic variants) that are likely to produce accurate predictions of ALS for a single individual, regardless of some possible limitations such as Male/Female ratio and age differences among the case and control groups. This study enrolled more than 50 medical cases with an accurate diagnosis of ALS and we were able to test them for 69 SNPs in 35 genes. Although the SALS patients analyzed represent a small cohort, it is nevertheless really representative from an epidemiological point of view (e.g. male/female ratio, bulbar/spinal ratio).

Besides, all ALS patients were previously screened for SOD1 gene mutations with negative results, thus confirming the sporadic nature of the disease. However, the sample size of 54 cases analysed for more than 60 SNPs, prompted us to look for valid, powerful and efficient statistical tools to approach and evaluate our data.

On the basis of the observed results some information related to the methodological approaches used can be assumed. The multiarray approach was previously validated by ourselves [17] and others [26] and contains TNF beta as the internal control.

Indeed, ApoE arg158cys was selected by all the five TWISTs while the ApoE cys112arg was selected only once. For NOS variants, the position -922 in the promoter region was never selected while the -690 variant in the promoter region too and the non synonymous variant in position 698 were both selected by all the five TWISTs. The two Factor VII and Selectin (SELE) genetic variants both containing the information necessary for the correct attribution to the disease vs healthy status, were selected five times (FVII arg353glu and SELE ser128arg) and four times (FVII del/ins and SELE leu554phe), respectively. The role of the paroxonase in predisposing to ALS disease appears to be confirmed: PON1 met 55leu and PON2 ser311cys were chosen four times, whereas PON1 gln192arg was never. PPARγ pro12ala was chosen four times: we can assume a generic role of this receptor on ALS disease since PPARγ is at the crossroads between lipid metabolism and innate immune response [34]. In addition, we noticed, for example, that in the same TNF locus, 6p21.3, lies also the HFE gene for hemocromatosis and the peripherin gene, both previously involved in ALS disease [35].

Few genetic variants were never selected by any of the TWIST procedures. One possible reason is that some information had already been picked up by the systems, e.g. for PON1, NOS and TNF. Moreover, regarding APOA4 and APO C3 variants we observed that they lie on chromosome 11 which may not be at all involved in the disease. Indeed, a very recent paper on genome wide genotyping in ALS [13], found no SNPs associated with the disease on chromosome 11.

From a biological point of view, the identified gene variations confirm some of the already known results (ApoE and PON for example) and identify new gene/genetic variations not known to be involved in the disease. Our results strengthen the involvement of oxidative stress as well as angiogenesis (NOS) and immune response (TNF) pathways. Besides, our results shed light on the involvement of lipid pathways (LIPC, PPARγ). Indeed, a role for polyunsaturated fatty acids has been postulated for the misfolding protein aggregations in several neurodegenerative diseases including familial ALS [36]. Furthermore polyunsaturated fatty acids could be enzymatically converted into various lipid mediators such as leukotriene and prostaglandins which have a strong biological activity in several signalling pathways [37].

## Conclusion

Our study has a major focus on disentangling the effect of interacting multiple low penetrance alleles on complex diseases. We analysed genetic variables within genes possibly involved in the ALS disease and thanks to artificial intelligence agents such as those employed in this study, on the basis of a subset of genetic data only, we were are able to conveniently differentiate ALS cases from control subjects. We still do not know which specific variation within the subset of SNP is linked to the disease, however ANNs are able to discriminate among cases and controls with only seven genetic SNPs.

We are aware that this is an exploratory study and that it should be replicated in another and much larger sample size, nevertheless this study offers new insight into genetic markers of sporadic ALS pointing out the existence of a strong genetic background. The data provide useful information to direct future research on the complexity of the genetic profile of ALS subjects.

## Authors' contributions

SP carried out the molecular genetic study, participated in the design of the study, coordinated and drafted the manuscript. EG participated in the design of the study, in the statistical analysis, coordinated and drafted the manuscript. MCP participated in genotyping. AM participated in conceiving the study. MB performed the statistical analysis and developed the intelligent systems. All authors read and approved the final manuscript
